# The Role of Gut Microbiota in Colorectal Cancer Pathogenesis: A Comprehensive Literature Review

**DOI:** 10.3390/ijms262411870

**Published:** 2025-12-09

**Authors:** Dan Nicolae Paduraru, Alexandru Cosmin Palcau, Valeriu Gabi Dinca, Diana Mihaela Ciuc, Alexandru Constantinescu

**Affiliations:** 1 Faculty of Medicine, Carol Davila University of Medicine and Pharmacy, 050474 Bucharest, Romania; dan.paduraru@umfcd.ro (D.N.P.); alexandru.constantinescu@umfcd.ro (A.C.); 2 Surgery Department, CF2 Clinical Hospital, 011464 Bucharest, Romania; dincagabi@yahoo.com; 3 IIIrd Clinic of General and Emergency Surgery, University Emergency Hospital, 050098 Bucharest, Romania; 4 Otolaryngology Department, CF2 Clinical Hospital, 011464 Bucharest, Romania; ciuc_diana@yahoo.com; 5 Gastroenterology Department, University Emergency Hospital, 050098 Bucharest, Romania

**Keywords:** colorectal cancer, gut microbiota, dysbiosis, carcinogenesis, inflammation, microbiome

## Abstract

Colorectal cancer (CRC) represents a significant global health burden, ranking as the third most frequently diagnosed malignancy worldwide. Emerging evidence has established a compelling association between gut microbiota dysbiosis and CRC pathogenesis, revealing complex mechanisms through which specific bacterial communities modulate carcinogenesis. This comprehensive review synthesizes current knowledge on the mechanistic contributions of gut microbiota to CRC development, with particular emphasis on key pathogenic bacteria including *Fusobacterium nucleatum*, *Bacteroides fragilis*, and *Escherichia coli*. We examine the molecular pathways through which these microorganisms promote tumorigenesis, including chronic inflammation induction, immune response modulation, metabolic reprogramming, and direct genotoxic effects. Furthermore, we discuss the therapeutic implications of microbiota-targeted interventions and the potential utility of microbial biomarkers for early CRC detection. Understanding the intricate host–microbiota interactions in CRC pathogenesis may facilitate the development of novel preventive strategies and therapeutic approaches for this devastating disease.

## 1. Introduction

Colorectal cancer constitutes a major oncological challenge globally, with its incidence steadily rising particularly in Western populations [1]. While genetic predisposition and environmental factors have traditionally been recognized as primary etiological contributors [2], the past decade has witnessed a paradigm shift in our understanding of CRC pathogenesis [3,4]. The human gastrointestinal tract harbors approximately 40 to 100 trillion microorganisms, collectively termed the gut microbiota, which play fundamental roles in maintaining intestinal homeostasis, modulating immune function, and mediating metabolic processes [5,6]. Mounting evidence suggests that perturbations in this microbial ecosystem, termed dysbiosis, represent a critical factor in colorectal tumorigenesis [7,8,9,10].

The westernization-driven alterations in dietary patterns and lifestyle have been implicated in modifying the gut microbial composition [11,12], potentially contributing to the increased CRC burden observed in industrialized nations [13,14]. The gut microbiota influences CRC development through multiple interconnected mechanisms, including the induction of chronic inflammation, regulation of host immune responses, production of genotoxic metabolites, and modulation of dietary component metabolism [15,16]. This review provides a comprehensive analysis of current knowledge regarding the mechanistic contributions of gut microbiota to CRC pathogenesis, with particular focus on recent advances in understanding the role of specific bacterial species and their potential as therapeutic targets and diagnostic biomarkers.

## 2. Gut Microbiota Composition and Dysbiosis in CRC

### 2.1. Normal Gut Microbiota Architecture

The healthy human gut microbiota exhibits remarkable diversity, comprising predominantly members of the phyla Firmicutes, Bacteroidetes, Actinobacteria, and Proteobacteria. This complex microbial community exists in a dynamic equilibrium, contributing to essential physiological functions including nutrient metabolism, vitamin synthesis, pathogen exclusion, and immune system education. The composition of gut microbiota varies longitudinally along the gastrointestinal tract and is influenced by numerous factors including host genetics, diet, age, medication use, and environmental exposures [17,18,19].

### 2.2. Dysbiotic Signatures in CRC

Clinical investigations have demonstrated consistent alterations in gut microbial composition associated with CRC development. Dysbiotic patterns observed in CRC patients typically manifest as decreased microbial diversity, reduced abundance of beneficial commensal bacteria, and enrichment of potentially pathogenic species [20,21]. Metagenomic analyses have identified specific microbial signatures that distinguish CRC patients from healthy controls, suggesting that microbiome profiling may serve as a non-invasive diagnostic approach [22,23].

The transition from normal colonic epithelium to adenomatous polyps and subsequently to invasive carcinoma is accompanied by progressive shifts in microbial community structure [24]. These changes are not merely consequences of tumor presence but actively participate in driving neoplastic transformation through various molecular mechanisms [25,26,27].

#### 2.2.1. Phylum- and Genus-Level Microbiota Alterations in CRC

Colorectal cancer is associated with profound and reproducible alterations in the gut microbiota, observable at both the phylum and genus levels. Metagenomic studies consistently show that CRC patients exhibit disrupted microbial community composition compared to healthy individuals. In the healthy gut, Firmicutes and Bacteroidetes normally dominate, making up roughly 90% of the microbiota, but in CRC this balance is significantly disturbed.

At the phylum level, the most notable change is the alteration of the Firmicutes/Bacteroidetes (F/B) ratio—an indicator of gut health and CRC risk. Both phyla progressively decline from healthy individuals through polyps and adenomas to full CRC, while Proteobacteria, Actinobacteria, Fusobacteria, and Verrucomicrobia become increasingly abundant. Proteobacteria shows the most dramatic rise across the carcinogenic sequence, suggesting increasing dysbiosis and inflammatory conditions conducive to tumor formation. Although Firmicutes remains the predominant phylum in preneoplastic and neoplastic tissue, it reaches its lowest levels in adenocarcinoma, whereas Bacteroidetes becomes more prominent, reversing the typical F/B ratio. Fusobacteria, although less abundant overall, is consistently enriched in CRC, and Verrucomicrobia is also significantly elevated, making it a potential marker of dysbiosis. Lower gastrointestinal tumors display a notably higher Bacteroidetes/Firmicutes ratio compared to upper GI tumors, highlighting site-specific microbial differences [28].

At the genus level, several bacteria are consistently enriched in CRC. *Fusobacterium*—especially *F. nucleatum*—is one of the strongest microbial signatures of CRC and appears early in tumorigenesis. Other genera often elevated in CRC include *Peptostreptococcus*, *Parvimonas*, *Gemella*, *Leptotrichia*, *Streptococcus*, *Lactobacillus*, *Bacteroides* (notably enterotoxigenic *B. fragilis*), and certain species of *Prevotella* and *Alistipes*. Many of these bacteria originate from the oral cavity and are normally poor colonizers of the healthy colon, but inflammation and neoplastic progression allow them to adhere to and invade colonic tissue [29].

In contrast, several beneficial genera decline markedly in CRC. These include *Faecalibacterium* (particularly the anti-inflammatory *F. prausnitzii*), *Roseburia*, and *Coprococcus*, all key butyrate producers essential for maintaining colonic health and anti-inflammatory balance. Members of the Lachnospiraceae family—also major butyrate producers—are characteristic of early lesions but diminish substantially in carcinomas, representing a significant functional loss in the CRC microbiome [30].

#### 2.2.2. Co-Abundance Groups and Microbial Interactions

Beyond individual taxa, CRC-associated dysbiosis includes changes in co-abundance groups (CAGs), indicating that microbial communities shift as interconnected networks. Specific CAGs involving Bacteroidetes, Firmicutes, and pathogen-rich clusters have been identified in CRC tissues. Network analyses highlight genera such as *Parvimonas*, *Peptostreptococcus*, *Prevotella*, *Alistipes*, *Odoribacter*, and *Butyricimonas* as central players in adenoma–carcinoma-associated microbial communities. These findings emphasize that CRC development involves coordinated ecosystem-level disruptions rather than isolated microbial changes [31].

Clinically, phylum- and genus-level microbial patterns offer valuable diagnostic and prognostic insights. These taxonomic shifts are consistent across diverse populations and also distinguish between upper and lower gastrointestinal tumors. Genus-level markers are particularly promising for clinical applications, as 16S rRNA sequencing—a widely used and cost-effective method—can reliably detect them. Understanding these broader taxonomic patterns also provides context for the involvement of known pathogenic species such as *F. nucleatum*, enterotoxigenic *B. fragilis*, and pks+ *E. coli*. Effective clinical interventions may therefore require a combined approach targeting specific pathogens while simultaneously restoring beneficial phyla and genera to re-establish a healthier microbial ecosystem [32].

## 3. Key Bacterial Species Implicated in CRC Pathogenesis

The selection of *Fusobacterium nucleatum*, enterotoxigenic *Bacteroides fragilis* (ETBF), and polyketide synthase-positive *Escherichia coli* (pks+ *E. coli*) as the primary focus of this review is based on several evidence-based criteria. First, these three bacterial species have demonstrated the most robust and reproducible associations with CRC across multiple independent cohorts and geographic populations, as evidenced by large-scale metagenomic studies. Second, each represents a distinct mechanistic paradigm of CRC pathogenesis: *F. nucleatum* exemplifies multi-factorial promotion through adhesion, inflammation, and immune evasion; ETBF demonstrates toxin-mediated carcinogenesis; and pks+ *E. coli* illustrates direct genotoxicity. Third, extensive mechanistic characterization in both in vitro cellular models and in vivo animal studies has established causal relationships between these bacteria and colorectal tumorigenesis, moving beyond mere association to demonstrate functional roles. Fourth, these organisms have been consistently identified through various complementary approaches including 16S rRNA sequencing, whole-genome shotgun metagenomic analysis, and functional screens for virulence factors. Finally, their potential as therapeutic targets and diagnostic biomarkers have been validated in preliminary clinical studies, making them particularly relevant for translational applications. While other bacterial species, including *Streptococcus gallolyticus*, *Peptostreptococcus anaerobius*, and certain *Prevotella* species, have also been implicated in CRC, the three organisms highlighted in this review possess the most comprehensive body of mechanistic and clinical evidence supporting their roles as key drivers of colorectal carcinogenesis.

### 3.1. Fusobacterium nucleatum

*Fusobacterium nucleatum* has emerged as one of the most extensively studied bacteria in CRC pathogenesis [33,34]. This Gram-negative anaerobe, typically residing in the oral cavity where it leads to dental plaque formation and periodontal disease [35,36], exhibits marked enrichment in colorectal tumors compared to adjacent normal tissue [37]. Recent genomic investigations have revealed subspecies-level variations in CRC association, with *F. nucleatum* subspecies *animalis* demonstrating particular dominance in the CRC niche through enhanced colonization capacity [38,39].

#### 3.1.1. Mechanisms of *F. nucleatum*-Mediated Carcinogenesis

Mounting evidence indicates that *F. nucleatum* exerts its pro-tumorigenic influence through a multifaceted interplay of molecular and cellular mechanisms that collectively promote tumor initiation, progression, and resistance to therapy (Table 1).

##### Adhesion and Invasion

One of the fundamental mechanisms by which *F. nucleatum* assists to colorectal carcinogenesis is through direct interaction with intestinal epithelial cells. The bacterium expresses a set of virulence-associated adhesins, among which Fusobacterium adhesin A (FadA) plays a pivotal role. FadA binds specifically to E-cadherin on host epithelial cells, disrupting cell–cell junction integrity and facilitating bacterial adhesion and invasion into the mucosal barrier [40]. This interaction activates downstream β-catenin and Wnt signaling cascades, resulting in enhanced transcription of oncogenic targets that drive cellular proliferation, inhibit apoptosis, and favor malignant transformation [34]. Furthermore, the breach of epithelial integrity by *F. nucleatum* allows microbial components and metabolites to interact with the subepithelial immune system, consequently amplifying local inflammation and genomic instability.

##### Inflammatory Response Induction

Chronic inflammation represents a well-established hallmark of colorectal tumorigenesis, and *F. nucleatum* has been shown to potentiate this process through the activation of innate immune receptors, particularly Toll-like receptors (TLRs). Upon recognition by TLR2 and TLR4, *F. nucleatum* triggers downstream NF-κB and MAPK signaling pathways, leading to upregulation of pro-inflammatory cytokines such as interleukin (IL)-6, IL-8, and tumor necrosis factor-alpha (TNF-α) [41]. The sustained secretion of these cytokines fosters a tumor-promoting milieu by recruiting myeloid-derived suppressor cells (MDSCs) and tumor-associated macrophages (TAMs), promoting angiogenesis, and facilitating epithelial–mesenchymal transition (EMT) [42]. This persistent inflammatory state not only accelerates neoplastic transformation but also plays a role in tumor immune tolerance and disease progression.

##### Immune Evasion

Beyond inflammation, *F. nucleatum* has evolved sophisticated mechanisms to circumvent host immune surveillance. The bacterial surface protein Fap2 interacts with the inhibitory receptor TIGIT expressed on natural killer (NK) cells and cytotoxic T lymphocytes (CTLs), leading to the suppression of anti-tumor cytotoxic responses [43]. By dampening NK- and T-cell–mediated cytolysis, *F. nucleatum* effectively shields tumor cells from immune-mediated elimination [44]. In addition, the bacterium has been implicated in modulating the tumor microenvironment toward an immunosuppressive phenotype, characterized by increased infiltration of regulatory T cells (Tregs) and altered cytokine signaling that collectively support immune escape.

##### Chemoresistance Promotion

Emerging data suggest that *F. nucleatum* not only initiates and sustains tumor growth but also confers resistance to chemotherapeutic agents. Mechanistically, the bacterium induces autophagy through activation of TLR4–MYD88 signaling, therefore reducing chemotherapy-induced apoptosis in colorectal cancer cells [45]. Moreover, *F. nucleatum* modulates the expression of specific microRNAs—such as miR-18a*, miR-4802, and miR-21—that regulate autophagy and apoptosis pathways, further enhancing chemoresistance [46]. Intriguingly, recent studies have demonstrated that *F. nucleatum* may translocate along with circulating tumor cells to distant metastatic sites, where it continues to promote proliferation and immune evasion, thus sustaining its oncogenic influence beyond the primary tumor [47,48]. This capacity for systemic dissemination underscores the bacterium’s potential role not only in tumor initiation but also in metastatic progression and recurrence.

Overall, these findings highlight the multifactorial nature of *F. nucleatum*-mediated carcinogenesis. By simultaneously targeting epithelial integrity, inflammatory signaling, immune modulation, and therapeutic response, *F. nucleatum* acts as both a driver and facilitator of colorectal tumor development and progression.

**Table 1:** The synthesis of *F. nucleatum* mechanism of carcinogenesis.

Mechanism	Description	Molecular/Cellular Effects	Consequences in Carcinogenesis
1. Adhesion and Invasion	*F. nucleatum* expresses surface adhesins (notably FadA) that bind to E-cadherin on intestinal epithelial cells.	Activates intracellular signaling pathways that promote cell proliferation and inhibit apoptosis.	Leads to uncontrolled epithelial cell growth and initiation of neoplastic transformation.
2. Induction of Inflammatory Response	Activates innate immune signaling pathways, including Toll-like receptors (TLRs).	Upregulates pro-inflammatory cytokines: IL-6, IL-8, TNF-α.	Creates a chronic inflammatory microenvironment conducive to tumor initiation and progression.
3. Immune Evasion	Suppresses anti-tumor immune surveillance through the surface protein Fap2.	Fap2 binds to the inhibitory receptor TIGIT on NK cells and T cells, inhibiting their cytotoxic activity.	Impairs immune-mediated tumor cell elimination, allowing tumor progression.
4. Promotion of Chemoresistance and Metastasis	Modulates cellular pathways involved in therapy response and tumor dissemination.	Induces autophagy, alters microRNA expression profiles; can translocate with tumor cells to distant sites.	Contributes to chemotherapy resistance and metastatic spread, maintaining pro-tumorigenic activity in secondary sites.

### 3.2. Bacteroides fragilis

*Bacteroides fragilis* is an anaerobic, Gram-negative commensal bacterium commonly residing in the human gut microbiota. Although typically considered a symbiont, certain strains—particularly enterotoxigenic *Bacteroides fragilis* (ETBF)—have been increasingly implicated in the initiation and progression of colorectal cancer (CRC). ETBF secretes a zinc-dependent metalloprotease toxin known as *B. fragilis* toxin (BFT, or fragilysin), which exerts profound effects on epithelial cell signaling, mucosal integrity, and host immune responses, thereby fostering a pro-tumorigenic microenvironment [49].

#### 3.2.1. BFT-Mediated Pathogenic Mechanisms

##### Epithelial Barrier Disruption and β-Catenin Pathway Activation

A central event in ETBF-induced tumorigenesis involves the proteolytic cleavage of E-cadherin, a critical adhesion molecule responsible for maintaining epithelial cell–cell junctions and structural cohesion of the intestinal barrier. BFT directly targets the extracellular domain of E-cadherin on colonic epithelial cells, disrupting adherens junctions and compromising epithelial integrity [50]. This disruption facilitates paracellular permeability and microbial translocation, exposing subepithelial immune cells to luminal antigens and microbial products. Moreover, E-cadherin cleavage releases β-catenin from its membrane-bound complex, leading to its nuclear translocation and activation of Wnt/β-catenin–dependent transcriptional programs. These signaling cascades upregulate oncogenic targets such as c-Myc and cyclin D1, driving uncontrolled cellular proliferation, resistance to apoptosis, and acquisition of neoplastic phenotypes [51]. The cumulative effect of these alterations is a shift from a homeostatic epithelial architecture toward a dysplastic and proliferative state that predisposes to malignant transformation.

##### Induction of Chronic Inflammation and Th17-Mediated Immune Responses

Beyond its direct epithelial effects, ETBF profoundly modulates the mucosal immune landscape. BFT stimulates the secretion of pro-inflammatory cytokines and chemokines, including interleukin (IL)-1β, IL-6, and IL-8, primarily through activation of nuclear factor κB (NF-κB) and signal transducer and activator of transcription 3 (STAT3) signaling pathways [49,52]. These mediators orchestrate a sustained inflammatory response characterized by recruitment of neutrophils, macrophages, and dendritic cells to the colonic mucosa. Importantly, ETBF selectively enhances the differentiation and expansion of T-helper 17 (Th17) cells through IL-6 and transforming growth factor-beta (TGF-β) signaling, leading to elevated production of IL-17 and IL-23 [45]. This Th17-driven immune response facilitates to a chronic inflammatory microenvironment that enhances epithelial proliferation, angiogenesis, and DNA damage, ultimately supporting tumor initiation and progression. In this context, IL-17 functions as a potent pro-tumorigenic cytokine that amplifies the inflammatory loop between epithelial and immune cells, further promoting neoplastic transformation [53].

##### Experimental Evidence and Tumorigenic Potential

The carcinogenic potential of ETBF has been extensively demonstrated in experimental animal models. In murine models, colonization with ETBF induces colitis characterized by epithelial hyperplasia, crypt abscess formation, and infiltration of inflammatory cells [54]. Prolonged colonization leads to the development of colonic tumors, confirming the bacterium’s direct contribution to tumorigenesis. These effects are largely dependent on BFT activity, as non-toxigenic *B. fragilis* strains fail to elicit comparable inflammatory or neoplastic changes [55]. Additionally, ETBF has been shown to synergize with other components of the gut microbiome, enhancing dysbiosis and facilitating the establishment of a pro-carcinogenic microbial ecosystem [56]. The interplay between microbial virulence factors, epithelial signaling, and host immunity thus represents a critical axis in ETBF-mediated colorectal carcinogenesis.

##### Integrated Pathophysiological Perspective

Collectively, *Bacteroides fragilis*—specifically its enterotoxigenic strains—contributes to colorectal tumorigenesis through a multifactorial mechanism encompassing epithelial injury, activation of oncogenic signaling pathways, and induction of chronic, Th17-dominated inflammation [57]. The combination of structural disruption of the epithelial barrier and immune-mediated tissue injury creates a self-perpetuating cycle of damage and regeneration, enhancing the risk of malignant transformation. This dual role—both as a direct epithelial modulator and as an immune activator—highlights *B. fragilis* as a pivotal microbial factor linking intestinal dysbiosis to colorectal cancer pathophysiology.

### 3.3. Escherichia coli

Certain strains of *Escherichia coli* (*E. coli*), particularly those harboring the polyketide synthase (pks) genomic island, have been increasingly recognized for their oncogenic potential in the context of colorectal cancer (CRC). The pks pathogenicity island encodes a complex biosynthetic machinery responsible for the production of colibactin, a hybrid nonribosomal peptide–polyketide genotoxin. This compound exerts direct DNA-damaging effects on host epithelial cells, linking bacterial infection and genomic instability through a mechanistic pathway that closely parallels classical mutagen-driven carcinogenesis [58,59].

#### 3.3.1. Colibactin Biosynthesis and Mechanism of Action

Colibactin is synthesized by the clb gene cluster within the pks island, comprising enzymes involved in nonribosomal peptide synthetase (NRPS) and polyketide synthase (PKS) functions. Once produced, colibactin is delivered into host cells via direct contact, where it alkylates DNA, inducing interstrand crosslinks and double-strand breaks (DSBs). These lesions activate the host DNA damage response, primarily through the ataxia-telangiectasia mutated (ATM) and p53 pathways, leading to cell cycle arrest, chromosomal aberrations, and replication stress. Incomplete or erroneous repair of these DSBs results in permanent mutations and genomic rearrangements, thus fueling the accumulation of oncogenic alterations [60,61]. Over time, chronic exposure to colibactin can overwhelm cellular repair mechanisms, fostering a mutational signature that resembles those found in human colorectal tumors [62]. Indeed, recent sequencing analyses have identified a specific “colibactin mutational footprint” in colorectal cancer genomes, underscoring its causal contribution to tumor initiation [63,64].

#### 3.3.2. Impact on Host Cellular Pathways and Genomic Instability

Beyond its direct genotoxic activity, pks+ *E. coli* also modulates key cellular signaling pathways associated with tumorigenesis. Colibactin exposure induces persistent activation of DNA damage checkpoints and stimulates senescence-associated secretory phenotypes (SASP), characterized by the release of pro-inflammatory cytokines such as IL-6 and IL-8 [65]. This inflammatory feedback not only supports tumor-promoting microenvironmental changes but also stimulates compensatory proliferation of neighboring uninjured cells, therefore amplifying the risk of malignant transformation. Furthermore, colibactin-induced DNA damage contributes to microsatellite instability and chromosomal copy-number alterations—two hallmark features of colorectal carcinogenesis [66]. The interplay between chronic DNA injury, inflammation, and cellular regeneration establishes a vicious cycle of mutagenesis and selection that accelerates neoplastic progression.

#### 3.3.3. Epidemiological and Experimental Evidence

Epidemiological studies have consistently demonstrated an increased prevalence of pks+ *E. coli* in the intestinal microbiota of patients with colorectal cancer compared to healthy controls, suggesting a strong association between colibactin production and tumor presence [67,68,69]. Notably, these strains are often enriched within mucosa-associated bacterial communities, particularly in the proximal colon, where they can establish close contact with epithelial cells. Experimental studies using mouse models have provided complementary evidence: colonization with pks+ *E. coli* facilitates DNA damage, dysplasia, and tumor formation, whereas colonization with isogenic pks^−^ mutants fails to reproduce these effects [60,70]. These findings collectively support a causative role for colibactin-producing *E. coli* in colorectal tumorigenesis.

#### 3.3.4. Synergistic Interactions Within the Tumor Microbiome

Emerging data also indicate that pks+ *E. coli* may act synergistically with other pathogenic members of the colorectal microbiota, such as *Fusobacterium nucleatum* and *Bacteroides fragilis*. The presence of these bacteria together can potentiate epithelial damage, exacerbate inflammation, and enhance DNA mutagenesis, thereby establishing a microbial consortium that collectively drives cancer-promoting processes. This ecological synergy suggests that microbial carcinogenesis in CRC is not the result of a single pathogen, but rather of coordinated interactions within a dysbiotic microbial network [71].

#### 3.3.5. Pathophysiological Implications

Taken together, these findings highlight the dual role of *E. coli* in colorectal carcinogenesis—as both a direct genotoxic agent and an indirect promoter of a tumor-permissive microenvironment. The production of colibactin links microbial metabolism to host genomic instability, bridging infection biology with cancer genetics. This genotoxin-driven mechanism provides a compelling example of how commensal bacteria can transition from benign colonizers to active participants in oncogenesis, emphasizing the need for targeted microbiome-based interventions in CRC prevention and therapy [67,72].

## 4. Molecular Mechanisms Linking Gut Microbiota to CRC

### 4.1. Chronic Inflammation

Chronic inflammation represents a fundamental mechanism through which gut microbiota supports CRC development [73]. Dysbiotic microbial communities induce sustained activation of pro-inflammatory signaling pathways, including nuclear factor kappa B (NF-κB) and signal transducer and activator of transcription 3 (STAT3) [74,75,76]. The resulting inflammatory milieu is characterized by elevated production of reactive oxygen species (ROS), reactive nitrogen species (RNS), and pro-inflammatory cytokines, all of which contribute to DNA damage, cellular proliferation, and inhibition of apoptosis [77,78].

The inflammatory response also enhances angiogenesis through upregulation of vascular endothelial growth factor (VEGF), providing necessary blood supply for tumor growth [79]. Moreover, inflammation-induced epithelial–mesenchymal transition (EMT) facilitates tumor invasion and metastasis [80].

### 4.2. Immune System Modulation

Gut microbiota exerts profound effects on both innate and adaptive immunity within the colonic microenvironment. Pathogenic bacteria can suppress anti-tumor immune responses while simultaneously promoting pro-tumorigenic inflammatory cells [81]. Specifically, certain bacterial species enhance recruitment and activation of myeloid-derived suppressor cells (MDSCs) and tumor-associated macrophages (TAMs), both of which inhibit cytotoxic T-cell function and create an immunosuppressive tumor microenvironment [82].

Furthermore, dysbiotic microbiota can impair the function of dendritic cells, compromising their ability to present tumor antigens and activate T-cell responses. This immunomodulatory capacity of gut bacteria has significant implications for cancer immunotherapy efficacy, as the microbiome composition influences responses to immune checkpoint inhibitors [83,84].

### 4.3. Metabolic Reprogramming

Gut microbiota participates in the metabolism of dietary components, producing a diverse array of metabolites that can either promote or inhibit carcinogenesis. The metabolic reprogramming observed in CRC involves enhanced glycolysis (Warburg effect) [85,86], increased fatty acid synthesis, lipogenesis, and glutaminolysis, processes that can be modulated by microbial metabolites [15,87].

#### 4.3.1. Harmful Microbial Metabolites

Certain bacterial species produce genotoxic or pro-inflammatory metabolites that contribute to CRC risk:

Secondary Bile Acids: Bacterial enzymes convert primary bile acids into secondary bile acids such as deoxycholic acid (DCA) and lithocholic acid (LCA), which exhibit cytotoxic and genotoxic properties, inducing oxidative stress and DNA damage [88,89].

Hydrogen Sulfide: Sulfate-reducing bacteria produce hydrogen sulfide, which inhibits butyrate oxidation in colonocytes and can damage DNA through oxidative mechanisms [90,91].

N-nitroso Compounds: Certain bacteria possess nitrosating activity, converting dietary nitrates and proteins into potentially carcinogenic N-nitroso compounds [92].

#### 4.3.2. Protective Microbial Metabolites

Conversely, commensal bacteria produce metabolites with anti-tumorigenic properties:

Short-Chain Fatty Acids (SCFAs): Beneficial bacteria ferment dietary fibers to produce SCFAs, particularly butyrate, acetate, and propionate [93]. Butyrate serves as the primary energy source for colonocytes and exhibits anti-inflammatory, anti-proliferative, and pro-apoptotic effects [94]. It acts as a histone deacetylase (HDAC) inhibitor, influencing gene expression patterns that suppress tumorigenesis [95].

Indole Derivatives: Tryptophan metabolism by commensal bacteria yields indole compounds that activate the aryl hydrocarbon receptor (AhR), promoting intestinal barrier integrity and anti-inflammatory responses [96].

### 4.4. Direct Genotoxic Effects

Beyond metabolite-mediated effects, certain bacterial species exert direct genotoxic effects on host cells through secreted toxins or virulence factors. As previously discussed, colibactin produced by pks+ *E. coli* and BFT from ETBF directly damage host DNA, contributing to mutational accumulation. *F. nucleatum* FadA adhesin activates β-catenin signaling, which advances cellular proliferation and oncogenic transformation [34,72,97].

### 4.5. Synergistic Interactions Among CRC-Associated Bacteria

While individual bacterial species exert distinct pathogenic effects, accumulating evidence demonstrates that microbial carcinogenesis in CRC involves complex synergistic interactions among multiple bacteria rather than isolated actions of single organisms. The tumor microenvironment supports a polymicrobial consortium in which different pathogenic species cooperate to amplify pro-tumorigenic signals, creating a microecological niche conducive to cancer progression.

#### 4.5.1. Evidence for Polymicrobial Cooperation

Clinical investigations consistently reveal that CRC-associated pathogenic bacteria rarely exist in isolation. Studies examining bacterial co-occurrence patterns in CRC tissues have identified that *F. nucleatum*, ETBF, and pks+ *E. coli* frequently coexist within the same tumor samples, with their combined presence associated with worse clinical outcomes compared to tumors harboring single bacterial species. Recent meta-analyses have demonstrated significant co-enrichment of *F. nucleatum* and *B. fragilis* in CRC patients, particularly in advanced-stage tumors, suggesting cooperative roles in disease progression [98].

The concept of an “oral-microbe-induced colorectal tumorigenesis model” has emerged, recognizing that bacteria originating from the oral cavity, including *F. nucleatum*, Peptostreptococcus stomatis, Parvimonas micra, and Porphyromonas gingivalis, often colonize CRC tissue as a polymicrobial community [99]. This observation suggests that bacterial translocation from oral sites to colorectal tumors may occur as coordinated communities rather than individual species, with collective functions exceeding the sum of individual contributions.

#### 4.5.2. Mechanistic Basis for Synergistic Pathogenesis

Inflammatory Amplification: The three key pathogenic bacteria examined in this review activate overlapping yet complementary inflammatory pathways. *F. nucleatum* strongly induces NF-κB signaling and pro-inflammatory cytokines including IL-6, IL-8, and TNF-α. ETBF specifically activates STAT3 signaling and facilitates Th17 responses with IL-17 production. While pks+ *E. coli* induces moderate inflammation through inflammasome activation, the DNA damage it inflicts triggers cellular stress responses that amplify inflammatory signals. When present simultaneously, these bacteria create a chronic inflammatory milieu more severe than that induced by any single species, establishing a self-reinforcing pro-tumorigenic environment [41,52,74].

Experimental studies demonstrate this synergistic effect. *F. nucleatum*-induced tumorigenesis in mouse models requires co-administration of pro-inflammatory agents such as dextran sodium sulfate (DSS), indicating that *F. nucleatum* synergizes with inflammatory stimuli from other sources. This finding suggests that inflammation induced by ETBF or other bacteria may provide the necessary inflammatory context for *F. nucleatum* to exert maximal tumorigenic effects [100]

##### Sequential and Complementary Genotoxicity

The temporal dynamics of bacterial involvement suggest a multi-stage model of microbial carcinogenesis. pks+ *E. coli* may function primarily during tumor initiation through direct DNA damage, creating mutational landscapes that predispose to transformation. Subsequently, *F. nucleatum* and ETBF contribute to tumor promotion and progression through sustained inflammation, immune evasion, and metabolic reprogramming. This sequential model is supported by observations that certain bacteria show stage-specific enrichment: pks+ *E. coli* may be more relevant in early adenoma formation, while *F. nucleatum* demonstrates progressive enrichment from adenoma to carcinoma and metastasis [101,102].

The genotoxic effects also exhibit complementarity. While colibactin induces DNA double-strand breaks with subsequent mutational burden, chronic inflammation generated by *F. nucleatum* and ETBF produces reactive oxygen species (ROS) that cause oxidative DNA damage. Additionally, BFT from ETBF, though primarily inflammatory, can induce DNA damage through indirect mechanisms. The combination of direct genotoxicity (colibactin), oxidative damage (inflammation-derived ROS), and toxin-mediated damage (BFT) creates multiple simultaneous insults to genomic integrity, accelerating malignant transformation [46,72].

##### Immune Evasion Networks

Each bacterial species employs distinct immune evasion strategies that, when combined, create a profoundly immunosuppressive tumor microenvironment. *F. nucleatum* inhibits NK cells and cytotoxic T cells through Fap2-TIGIT interactions, effectively blocking cell-mediated anti-tumor immunity [43]. ETBF modulates dendritic cell function and promotes regulatory T cell responses, impairing antigen presentation and T cell priming [103]. While pks+ *E. coli* exhibits limited direct immune suppression, the DNA damage inflicted can activate immunosuppressive pathways including those involving myeloid-derived suppressor cells (MDSCs) [104]. The cumulative effect creates multiple layers of immune suppression operating through independent mechanisms, making it extremely difficult for the host immune system to mount effective anti-tumor responses.

##### Metabolic Cooperation

Bacterial metabolic activities within the tumor microenvironment demonstrate cooperative interactions. *F. nucleatum* alters tumor metabolism and stimulates autophagy, potentially creating metabolic conditions favorable for other bacterial species. The chronic inflammation induced by ETBF alters cellular metabolism, enhancing glycolysis (Warburg effect) and creating acidic, hypoxic conditions that may favor anaerobic bacteria including *F. nucleatum* itself [85,86]. Additionally, metabolic byproducts from one bacterial species may serve as nutrients or signaling molecules for others, establishing metabolic cross-feeding relationships that stabilize the polymicrobial community.

##### Biofilm Formation and Spatial Organization

*F. nucleatum* functions as a “bridging organism” in polymicrobial biofilms, possessing surface adhesins that facilitate aggregation of diverse bacterial species [105]. This capacity likely extends to the tumor microenvironment, where *F. nucleatum* may organize polymicrobial communities in spatial structures that enhance colonization persistence and resistance to immune clearance and antimicrobial treatments. Biofilm-associated bacteria exhibit altered gene expression patterns and increased resistance to environmental stresses compared to planktonic counterparts, potentially amplifying pathogenic activities [106].

#### 4.5.3. Clinical and Translational Implications

Recognition of polymicrobial synergism has profound implications for therapeutic strategies. Single-target antimicrobial approaches may prove insufficient if they eliminate one pathogen while leaving others intact to maintain the pro-tumorigenic microenvironment. Multi-targeted strategies simultaneously addressing multiple bacterial species may be necessary for effective intervention. Alternatively, disrupting key bacterial interactions—such as targeting *F. nucleatum*’s bridging function in polymicrobial communities—might destabilize entire pathogenic consortia.

Diagnostic approaches should similarly consider microbial communities rather than individual species. Combined detection of multiple CRC-associated bacteria may provide superior diagnostic accuracy and prognostic value compared to single-biomarker approaches. Studies have demonstrated that the co-presence of *F. nucleatum* and *B. fragilis* correlates with advanced disease stage and worse survival outcomes, suggesting that polymicrobial signatures represent more robust clinical markers.

Future research priorities include: (1) comprehensive characterization of polymicrobial community structures in CRC using spatial metagenomics and single-cell approaches; (2) experimental studies examining bacterial co-infections in animal models to dissect synergistic mechanisms; (3) clinical trials evaluating combination antimicrobial or microbiota-modulating strategies targeting multiple pathogenic species; and (4) development of systems biology models integrating host-bacteria and bacteria-bacteria interactions to predict therapeutic responses.

## 5. Gut Microbiota as Diagnostic Biomarkers

The consistent microbial alterations observed in CRC have prompted investigation into the utility of gut microbiota profiling as a non-invasive diagnostic tool [107]. Current CRC screening methods, including fecal immunochemical tests (FIT) and colonoscopy, face limitations regarding sensitivity, specificity, cost, and patient compliance. Microbiome-based diagnostics offer potential advantages as non-invasive, cost-effective alternatives for early CRC detection and risk stratification [108].

Advanced molecular techniques including 16S rRNA gene sequencing, metagenomic shotgun sequencing, and metabolomic profiling have enabled comprehensive characterization of gut microbial communities [105,109]. Machine learning algorithms applied to microbiome data have demonstrated promising accuracy in distinguishing CRC patients from healthy controls and detecting precancerous lesions [110,111]. Specific bacterial signatures, including elevated abundance of *F. nucleatum*, ETBF, and pks+ *E. coli*, combined with reduced diversity of beneficial species, show potential as diagnostic biomarkers [71,112].

### Methods for Bacterial Detection and Characterization

The identification and quantification of CRC-associated bacteria employ multiple complementary methodological approaches, each with distinct advantages and limitations. 16S rRNA gene sequencing remains the most widely utilized method for taxonomic profiling of gut microbial communities. This culture-independent technique involves PCR amplification of the bacterial 16S ribosomal RNA gene, followed by next-generation sequencing to identify bacterial taxa based on sequence homology to reference databases. While 16S sequencing provides cost-effective community-level characterization, its resolution is limited to genus or species level, and it cannot detect specific virulence factors such as the pks island or BFT toxin genes.

Whole-genome shotgun metagenomic sequencing overcomes these limitations by sequencing total microbial DNA, enabling species-level resolution and functional gene detection. This approach can identify specific virulence determinants, including the pks genomic island in *E. coli*, the bft gene in *B. fragilis*, and adhesin-encoding genes in *F. nucleatum*. However, metagenomic sequencing requires greater sequencing depth and computational resources, increasing cost and analytical complexity [105].

Quantitative PCR (qPCR) targeting species-specific genes or virulence factors provides sensitive and specific quantification of particular bacteria. Multiplex qPCR assays have been developed to simultaneously detect *F. nucleatum*, ETBF, and pks+ *E. coli* in clinical samples. Fluorescence in situ hybridization (FISH) enables spatial localization of bacteria within tissue samples, providing information about bacterial proximity to tumor cells and penetration into tumor architecture, which has proven particularly valuable for *F. nucleatum* studies [109].

Regarding biomaterials, fecal samples represent the most commonly used non-invasive specimen for gut microbiome analysis. Stool samples are easily collected, contain high bacterial loads, and reflect the luminal microbiota composition. Standardized collection protocols using preservation buffers or immediate freezing maintain sample integrity for molecular analysis. Multiple studies have successfully identified CRC-associated microbial signatures in fecal samples, demonstrating the utility of stool-based diagnostics.

Mucosal biopsies and surgical tissue specimens provide information about the mucosa-associated microbiota, which may differ substantially from luminal populations. Tissue samples enable direct assessment of bacterial invasion into tumor tissue and spatial analysis via FISH or immunohistochemistry. Studies comparing tumor tissue with adjacent normal mucosa have revealed enrichment of *F. nucleatum* specifically within tumor tissue, supporting its role in tumor progression [96,97,98,99,100,101,102,103,104,105,106,107,108,109,110,111,112].

Blood samples have limited utility for direct bacterial detection in CRC, as bacteremia is typically not a feature of CRC-associated dysbiosis under normal circumstances. However, serological approaches detecting antibodies against CRC-associated bacteria, such as anti-*F. nucleatum* antibodies, have shown potential as diagnostic biomarkers. Additionally, circulating bacterial DNA or extracellular vesicles derived from gut bacteria can occasionally be detected in plasma using highly sensitive molecular techniques, though this remains primarily a research tool rather than a clinical diagnostic approach.

Saliva samples have demonstrated utility specifically for *F. nucleatum* detection, given its oral origin. Studies have shown correlations between salivary and colorectal tumor *F. nucleatum* abundance, suggesting potential for non-invasive screening, though this approach requires further validation.

The choice of biomaterial and detection method should be guided by the specific research question or clinical application, with fecal samples and molecular sequencing-based approaches representing the most practical combination for microbiome-based CRC diagnostics in clinical settings.

However, several challenges must be addressed before clinical implementation, including inter-individual variability in microbiome composition, geographic and ethnic differences in microbial profiles, standardization of sample collection and processing methods, and validation across diverse populations.

The comparative analysis of the key pathogenic microorganisms in colorectal cancer is presented in Table 2.

## 6. Therapeutic Implications

### 6.1. Microbiota-Targeted Interventions

The recognition of gut microbiota as a critical player in CRC pathogenesis has opened new avenues for therapeutic intervention:

Probiotics: Administration of beneficial bacterial strains may restore microbial balance and inhibit pathogenic species. Specific probiotic strains have demonstrated anti-inflammatory, immunomodulatory, and anti-proliferative effects in preclinical studies [113].

Prebiotics: Non-digestible dietary fibers that selectively promote growth of beneficial bacteria, particularly SCFA-producing species, may reduce CRC risk through enhancement of protective metabolite production [114].

Synbiotics: Combined administration of probiotics and prebiotics may provide synergistic benefits in modulating gut microbiota composition and function [115].

Fecal Microbiota Transplantation (FMT): Transfer of fecal material from healthy donors to CRC patients represents a more comprehensive approach to microbiota restoration, though its safety and efficacy in CRC context require further investigation [116].

Antibiotics: Selective antimicrobial strategies targeting specific pathogenic bacteria, such as *F. nucleatum*, have shown promise in preclinical models for reducing tumor burden and enhancing therapeutic responses [117].

#### 6.1.1. Selective Antimicrobial Strategies Targeting CRC-Associated Bacteria

The development of selective antimicrobial approaches targeting specific pathogenic bacteria while preserving beneficial commensal microbiota represents a promising therapeutic strategy for CRC prevention and treatment. Unlike broad-spectrum antibiotics that indiscriminately eliminate both pathogenic and protective bacteria, potentially exacerbating dysbiosis, precision antimicrobial strategies aim to selectively eliminate or suppress key CRC-promoting organisms.

##### Targeted Antibiotic Approaches for *F. nucleatum*

*Fusobacterium nucleatum* exhibits susceptibility to several antibiotic classes, with metronidazole demonstrating particularly potent activity against this anaerobic organism. Preclinical studies in mouse models have shown that metronidazole treatment reduces *F. nucleatum* colonization in colorectal tumors and decreases tumor burden, particularly when combined with conventional chemotherapy [118]. Additionally, *F. nucleatum* shows sensitivity to β-lactam antibiotics, including amoxicillin and ampicillin, as well as to clindamycin. Combination regimens using metronidazole with a β-lactam antibiotic may provide synergistic effects while minimizing resistance development. Recent research has explored pulsed antibiotic treatment protocols that periodically eliminate *F. nucleatum* without causing sustained broad-spectrum dysbiosis, though optimal dosing and timing require further clinical investigation [119].

##### Targeting ETBF

Enterotoxigenic *B. fragilis* is generally susceptible to metronidazole, carbapenems (such as imipenem and meropenem), and β-lactam/β-lactamase inhibitor combinations (such as piperacillin-tazobactam). However, Bacteroides species can harbor multiple antibiotic resistance genes, and increasing resistance to commonly used antibiotics has been documented. Alternative approaches focus on neutralizing *B. fragilis* toxin rather than eliminating the bacteria entirely. Toxin-neutralizing monoclonal antibodies and small molecule inhibitors of BFT metalloprotease activity are under development, offering the potential to block pathogenic effects without disturbing overall microbiota composition [120].

##### Strategies Against pks+ *E. coli*

While *E. coli* is typically susceptible to fluoroquinolones, aminoglycosides, and third-generation cephalosporins, the high prevalence of antibiotic resistance among *E. coli* strains complicates targeted elimination strategies. Moreover, non-pathogenic *E. coli* strains constitute part of the normal microbiota, making selective targeting of only pks+ strains challenging. Innovative approaches focus on inhibiting colibactin biosynthesis rather than bacterial elimination. Small molecule inhibitors targeting the ClbP peptidase, essential for colibactin maturation and export, have shown promise in preclinical models, successfully blocking genotoxic effects without killing the bacteria [121]. This strategy preserves the ecological niche occupied by *E. coli*, preventing colonization by potentially more harmful organisms.

##### Bacteriophage Therapy

Bacteriophages offer exquisite specificity, with individual phage strains capable of targeting specific bacterial species or even strains. Phage therapy targeting *F. nucleatum* has been explored in preclinical studies, demonstrating successful bacterial elimination with minimal effects on other microbiota members. Engineered phages carrying genes encoding antimicrobial peptides or biofilm-disrupting enzymes may enhance efficacy. Phage cocktails targeting multiple CRC-associated pathogens simultaneously could provide comprehensive therapeutic intervention. However, phage therapy faces regulatory and manufacturing challenges that must be addressed before clinical implementation [122].

##### Antimicrobial Peptides and Bacteriocins

Naturally occurring antimicrobial peptides produced by beneficial bacteria (bacteriocins) demonstrate selectivity for closely related bacterial species. Bacteriocins produced by probiotic bacteria have shown activity against pathogenic species while sparing more distantly related commensals. Engineering probiotics to produce bacteriocins with enhanced activity against *F. nucleatum*, ETBF, or pks+ *E. coli* represents an innovative approach combining probiotic benefits with targeted pathogen suppression [123].

##### Challenges and Future Directions

Several challenges must be addressed for clinical translation of selective antimicrobial strategies. These include: (1) developing reliable methods to identify patients harboring specific pathogenic bacteria requiring treatment; (2) determining optimal timing, dosing, and duration of antimicrobial interventions; (3) preventing resistance development through judicious use and combination approaches; (4) assessing long-term effects on microbiome stability and function; and (5) establishing safety and efficacy through rigorous clinical trials. Despite these challenges, selective antimicrobial targeting of CRC-associated pathogenic bacteria represents a promising avenue for precision medicine approaches in CRC prevention and treatment, particularly as companion strategies to conventional therapies or in high-risk populations for primary prevention.

### 6.2. Enhancement of Cancer Therapy Efficacy

Accumulating evidence indicates that gut microbiota composition influences responses to conventional cancer therapies and immunotherapy. Specific bacterial species can modulate chemotherapy efficacy through effects on drug metabolism, immune activation, and intestinal barrier integrity [45,46]. Furthermore, the microbiome profoundly impacts responses to immune checkpoint inhibitors, with certain beneficial bacterial species promoting anti-tumor immunity while pathogenic bacteria may impair immunotherapy efficacy [15].

Optimization of gut microbiota composition through dietary interventions, probiotic supplementation, or FMT prior to or concurrent with cancer treatment may improve therapeutic outcomes and reduce treatment-related toxicities. This represents an emerging field with significant clinical potential requiring rigorous investigation in clinical trials.

## 7. Future Directions and Challenges

Despite remarkable advances in elucidating the relationship between the gut microbiota and colorectal cancer (CRC), numerous challenges remain that hinder full translation of microbiome science into clinical oncology. Future research must move beyond descriptive correlations to mechanistic and translational frameworks that clarify causality, temporal evolution, and host–microbe interactions while paving the way for precision-based preventive and therapeutic strategies.

Although robust associations between specific bacteria—such as *Fusobacterium nucleatum*, enterotoxigenic *Bacteroides fragilis* (ETBF), and pks+ *Escherichia coli*—and CRC have been repeatedly demonstrated, establishing definitive causality remains a critical challenge. The central question persists: are these microorganisms true “drivers” of tumorigenesis or merely “passengers” that flourish within the tumor microenvironment? Addressing this question requires refined experimental designs integrating gnotobiotic animal models, human organoid systems, and longitudinal patient cohorts. Mechanistic studies using targeted microbial knockouts, metabolomic profiling, and host-pathway analyses will be indispensable to dissect the specific bacterial effectors, signaling cascades, and host responses that directly contribute to carcinogenesis. Establishing causality will also necessitate the identification of reproducible microbial “oncogenic signatures” that can distinguish pathogenic colonization from incidental microbial shifts secondary to tumor-associated dysbiosis [124].

Another major gap in knowledge involves the temporal dimension of microbiota-mediated carcinogenesis. Most existing data are cross-sectional, capturing static snapshots of microbial composition at a single disease stage. Longitudinal studies that follow individuals from healthy mucosa through adenomatous stages to invasive carcinoma are essential to map the dynamic evolution of microbial communities during tumor initiation and progression. Such studies will help delineate critical “windows of susceptibility” during which microbial dysbiosis exerts maximal oncogenic influence and may identify early microbial biomarkers predictive of neoplastic transformation. Integration of temporal microbiome profiling with histopathological and molecular tumor features could reveal progressive shifts in bacterial metabolism, virulence gene expression, and host inflammatory signaling—offering novel targets for early intervention and surveillance [125].

Traditional microbiome studies have largely relied on 16S rRNA sequencing or metataxonomic profiling, which, while informative, provide limited insight into functional potential. A major future direction lies in expanding to functional metagenomics, transcriptomics, and metabolomics to capture the true biochemical and genetic capabilities of the microbiota. This shift from “who is there” to “what they are doing” will allow identification of microbial metabolites, virulence factors, and signaling molecules that directly modulate host pathways. Multi-omics integration—linking metagenomic data with transcriptomic, proteomic, and metabolomic readouts—will enable a systems-level understanding of microbial contributions to carcinogenesis [126]. Furthermore, mapping microbial gene expression within tumor versus adjacent normal tissues could help pinpoint microbial activities specifically associated with the neoplastic niche.

CRC development reflects not only microbial composition but also the host’s intrinsic susceptibility. Genetic polymorphisms, immune competence, metabolic status, and diet profoundly shape the gut ecosystem and determine the host’s response to microbial stimuli [127]. Future studies should adopt integrative multi-omics approaches—combining host genomics, transcriptomics, and immunophenotyping with microbiome and metabolome profiling—to unravel the bidirectional crosstalk between microbes and host. For instance, elucidating how host immune checkpoints, epithelial barrier integrity, or mucosal glycan expression modulate bacterial colonization and virulence could clarify the interdependence between host genotype and microbial pathogenicity [128]. Such insights will be crucial to understand why certain individuals develop CRC in response to dysbiosis while others remain unaffected despite similar microbial exposures.

Translating microbiome discoveries into clinically actionable tools remains a formidable challenge. The development of microbiota-based diagnostics, prognostic biomarkers, and therapeutic interventions requires rigorous validation through multicenter clinical trials with standardized sampling, sequencing, and analytical protocols. The heterogeneity of patient populations—across geography, diet, genetics, and healthcare systems—must be accounted for to ensure reproducibility and generalizability of results. Moreover, regulatory and ethical frameworks for microbial manipulation (e.g., fecal microbiota transplantation, engineered probiotics, or targeted bacteriophage therapy) will need to evolve in parallel with scientific advances. Only through standardized methodologies and harmonized biobanking will microbiome research achieve the consistency necessary for clinical implementation.

Ultimately, the integration of microbiome science into precision oncology represents a transformative frontier. Personalized strategies that tailor prevention and treatment based on an individual’s microbial, genetic, and immunologic profile could revolutionize CRC management. Microbiota-targeted interventions—ranging from dietary modulation and prebiotics to selective antimicrobial agents and next-generation probiotics—may be optimized to restore microbial balance and mitigate oncogenic risk. In addition, predictive algorithms combining microbial biomarkers with host molecular features could enable risk stratification and individualized surveillance protocols. The convergence of microbiome research with artificial intelligence, computational modeling, and personalized medicine promises to shift the paradigm from population-based screening to microbiota-informed precision prevention.

## 8. Integrating Microbiota into the Framework of CRC Heterogeneity

Colorectal cancer represents a heterogeneous disease, exhibiting substantial variability in molecular features, clinical presentation, therapeutic response, and patient outcomes. The recognition of this heterogeneity has driven the development of molecular classification systems, most notably the Consensus Molecular Subtypes (CMS) framework, which stratifies CRC into four distinct subtypes based on transcriptomic profiles: CMS1 (microsatellite instability immune subtype), CMS2 (canonical epithelial subtype), CMS3 (metabolic subtype), and CMS4 (mesenchymal subtype). Each CMS subtype exhibits unique biological characteristics, prognostic implications, and therapeutic sensitivities. Critically, emerging evidence demonstrates that gut microbiota composition and microbial pathogenesis patterns are not uniform across CRC but instead display CMS-specific associations, indicating that microbial carcinogenesis must be understood within the broader context of tumor molecular heterogeneity [129].

The ultimate goal is comprehensive systems biology integration that simultaneously considers host genetics, somatic mutations, transcriptomics, epigenomics, tumor microenvironment characteristics, and microbiome composition. Advanced computational approaches including machine learning, network analysis, and multi-omics integration are enabling such holistic perspectives. Recent studies employing integrative multi-omics clustering with microbiome data have identified novel CRC subtypes with enhanced prognostic value and immunotherapy response prediction beyond traditional CMS classification.

## 9. Conclusions

The gut microbiota has emerged as a critical modulator of CRC pathogenesis, operating not as isolated bacterial species but as complex polymicrobial communities that synergistically promote carcinogenesis through inflammation induction, immune response regulation, metabolic modulation, and direct genotoxic effects. Key bacterial species, particularly *F. nucleatum*, ETBF, and pks+ *E. coli*, exert distinct yet complementary pathogenic mechanisms that collectively establish pro-tumorigenic microenvironments.

Critically, microbial contributions to CRC must be understood within the framework of tumor molecular heterogeneity. The association of specific microbial signatures with Consensus Molecular Subtypes demonstrates that microbiota-driven carcinogenesis is not uniform but exhibits subtype-specific patterns reflecting bidirectional interactions between microbial communities and tumor molecular landscapes. This recognition necessitates integration of microbiome profiling with molecular classification for optimal risk stratification and therapeutic selection.

The consistent microbial alterations observed in CRC offer promise for development of non-invasive diagnostic biomarkers and novel therapeutic strategies targeting the microbiome. However, effective interventions must address polymicrobial communities rather than individual species, recognizing synergistic interactions that amplify pathogenic effects. Multi-targeted antimicrobial strategies, microbiota restoration approaches, and integration of microbiome-modulating interventions with conventional therapies represent promising avenues for improving clinical outcomes.

As our understanding of host–microbiota interactions in CRC continues to evolve through advanced molecular techniques, mechanistic investigations, and rigorously designed clinical trials, integration of microbiome science into precision oncology holds significant potential for transforming CRC prevention, early detection, risk stratification, and treatment. The paradigm shift recognizing gut microbiota as critical players in CRC pathogenesis within the context of tumor heterogeneity represents a transformative advancement in oncology, opening new frontiers that may ultimately reduce the global burden of colorectal cancer.

## Figures and Tables

**Table 2:** Summary Comparison of The Three Major CRC-Associated Bacteria.

Feature	*Fusobacterium nucleatum*	*Bacteroides fragilis* (ETBF)	*Escherichia coli* (pks+)
Primary Mechanism	Multi-factorial: adhesion, inflammation, immune suppression	Toxin-mediated inflammation and barrier disruption	Direct DNA damage via colibactin genotoxin
Key Virulence Factor	FadA adhesin, Fap2 protein	*B. fragilis* toxin (BFT) metalloprotease	Colibactin (polyketide genotoxin)
Target	E-cadherin, immune cells (NK, T cells)	E-cadherin, epithelial tight junctions	Host cell DNA
Inflammatory Response	Strong (IL-6, IL-8, TNF-α, NF-κB)	Moderate-Strong (Th17, IL-17, STAT3)	Mild-Moderate (IL-1β, IL-6)
Genotoxicity	Indirect (via ROS)	Minimal	Direct (DNA double-strand breaks)
Immune Evasion	High (inhibits NK and T cells via Fap2-TIGIT)	Moderate (modulates dendritic cells, Tregs)	Low
Role in CRC Stages	Adenoma through metastasis	Early inflammation and adenoma formation	Tumor initiation (DNA damage)
Chemoresistance	Yes (autophagy, microRNA modulation)	Possible (via chronic inflammation)	Limited evidence
Metastatic Potential	High (translocates with tumor cells)	Low	Minimal
Main Signaling Pathways	Wnt/β-catenin, NF-κB, TLR4	Wnt/β-catenin, NF-κB, STAT3	ATM/ATR, DNA damage response, p53
Biomarker Utility	High (diagnostic and prognostic)	Moderate (risk stratification)	Moderate (requires pks detection)
Therapeutic Strategy	Antibiotics, adhesin inhibitors, immunotherapy	Toxin neutralization, antibiotics	Colibactin inhibitors, DNA repair enhancement

## Data Availability

No new data were created or analyzed in this study.
